# Combining TIR and FRET in Molecular Test Systems

**DOI:** 10.3390/ijms20030648

**Published:** 2019-02-02

**Authors:** Herbert Schneckenburger, Petra Weber, Michael Wagner, Sandra Enderle, Bernd Kalthof, Linn Schneider, Claudia Herzog, Julian Weghuber, Peter Lanzerstorfer

**Affiliations:** 1 Institute of Applied Research, Aalen University, 73430 Aalen, Germany; Petra.Weber@hs-aalen.de (P.W.); Michael.Wagner@hs-aalen.de (M.W.); sandra-enderle@gmx.de (S.E.); 2 Pharmaceutical Division, Bayer AG, 42096 Wuppertal, Germany; bernd.kalthof@bayer.com (B.K.); linn.schneider@bayer.com (L.S.); claudia.herzog@bayer.com (C.H.); 3 University of Applied Sciences Upper Austria, 4600 Wels, Austria; Julian.Weghuber@fh-wels.at (J.W.); Peter.Lanzerstorfer@fh-wels.at (P.L.); 4 Austrian Competence Center for Feed and Food Quality, Safety and Innovation, 4600 Wels, Austria

**Keywords:** microscopy, living cells, fluorescence spectroscopy, fluorescence lifetime imaging (FLIM), total internal reflection (TIR), Förster resonance energy transfer (FRET), fluorescence reader, drug screening

## Abstract

Pharmaceutical agents or drugs often have a pronounced impact on protein-protein interactions in cells, and in particular, cell membranes. Changes of molecular conformations as well as of intermolecular interactions may affect dipole-dipole interaction between chromophoric groups, which can be proven by measuring the Förster resonance energy transfer (FRET). If these chromophores are located within or in close proximity to the plasma membrane, they are excited preferentially by an evanescent electromagnetic wave upon total internal reflection (TIR) of an incident laser beam. For the TIR-FRET screening of larger cell collectives, we performed three separate steps: (1) setting up of a membrane associated test system for probing the interaction between the epidermal growth factor receptor (EGFR) and the growth factor receptor-bound protein 2; (2) use of the Epac-SH188 sensor for quantitative evaluation under the microscope; and (3) application of a TIR fluorescence reader to probe the interaction of GFP with Nile Red. In the first two steps, we measured FRET from cyan (CFP) to yellow fluorescent protein (YFP) by spectral analysis and fluorescence lifetime imaging (FLIM) upon illumination of whole cells (epi-illumination) as well as selective illumination of their plasma membranes by TIR. In particular, TIR excitation permitted FRET measurements with high sensitivity and low background. The Epac sensor showed a more rapid response to pharmaceutical agents, e.g., Forskolin or the A2B adenosine receptor agonist NECA, in close proximity to the plasma membrane compared to the cytosol. Finally, FRET from a membrane associated GFP to Nile Red was used to test a multi-well TIR fluorescence reader with simultaneous detection of a larger number of samples.

## 1. Introduction

Measurement of Förster resonance energy transfer (FRET) [1] between a donor and an acceptor molecule (intermolecular FRET) or between different chromophoric groups of a larger molecule, e.g., a protein (intramolecular FRET), has become a valuable tool for probing either molecular interactions or conformational changes of a molecule in the nanometer range. The method is based on optical excitation of a so-called donor molecule and interaction of optical transition dipoles with an acceptor molecule, which is able to fluoresce. Due to the impact of chemical or pharmaceutical agents, the FRET technique is increasingly applied in biosensors and drug discovery systems [2,3,4,5]. FRET measurements of living cells have been reported for about 30 years [6,7], but recently their plasma membranes have been assessed selectively, e.g., upon total internal reflection (TIR) of a laser beam with an evanescent electromagnetic field penetrating a small distance into the cell [2,8,9,10,11]. Selective examination of the plasma membrane and adjacent cellular sites excludes superimposing signals from the cytoplasm or the supernatant and permits measurement at the interface of a cell with its environment, including the interactions of molecules with ion channels [12,13] as well as protein-protein interactions, which may play a role in the pathogenesis of various diseases [14,15,16,17]. While TIR-FRET measurements reported in the literature are commonly focused on small molecular assemblies (see above) or even single molecules [18,19,20], it is our aim to develop a test system that is appropriate for larger cell collectives using high content or even high throughput screening techniques. Therefore, high transfection rates, larger numbers of fluorophores as well as specific microscope and reader technologies are required. We attempted achieve our goal in three steps: (1) setting up of an appropriate test system with membrane associated EGFR-CFP and Grb2-YFP, (2) use of the Epac-SH188 sensor for quantitative evaluation under the microscope, and (3) application of a TIR fluorescence reader to probe the interaction of GFP with Nile Red.

**Figure 1:**
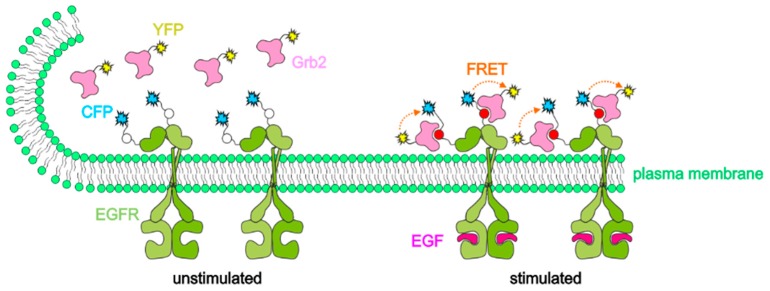
Förster resonance energy transfer (FRET) measurement for probing the proximity of EGFR-CFP and Grb2-YFP fusion proteins, which is favored by binding of the epidermal growth factor, EGF (“stimulation”).

First, we report on TIR-FRET measurements of the interaction between the epidermal growth factor receptor (EGFR) and the growth factor receptor-bound protein 2 (Grb2). The EGFR regulates important pathways such as growth, survival, proliferation and differentiation in mammalian cells, and it has become a major drug target as EGFR signaling is critical for the development of many types of cancer [21,22,23]. Therefore, it is necessary to develop new assays that facilitate the identification of novel EGFR modulators in a live cell context at sufficient throughput rates. For TIR-FRET measurements, the EGFR is fused with cyan fluorescent protein (CFP) and Grb2 with yellow fluorescent protein (YFP), as depicted in Figure 1. Hereby, FRET from CFP to YFP is examined, and compared to the whole cell experiments reported in the literature [24,25,26], TIR experiments were more selective for detection of membrane associated fluorophores. In addition to intensity measurements of donor and acceptor fluorescence, measurements of the fluorescence lifetime τ of the donor molecule (CFP) in the nanosecond range are expected to give reliable results, since they do not need any standard or calibration. τ is the reciprocal of all rates of deactivation of an excited molecular state, and if a lifetime τ_0_ is determined for a reference system without FRET, the difference between 1/τ and 1/τ_0_ reflects the rate of energy transfer k_ET_ according to

k_ET_ = 1/τ − 1/τ_0_ (1)

TIR experiments also appear to be applicable to intracellular molecules, e.g., fluorescent proteins, if their expression level is high enough, and parts of them are located in close proximity to the plasma membrane, since in this case, the background is also reduced considerably in comparison with the measurements of whole cells. An example is the so-called Epac-SH188 sensor (exchange protein directly activated by cAMP) for cyclic adenosine monophosphate [27,28], where upon addition of certain metabolites the conformation of the Epac molecule changes, and dipole-dipole interaction between chromophoric groups (CFP and YFP) is modified. In the present study, reaction kinetics in close proximity to the plasma membrane (assessed by TIR) and inside the cells (assessed by whole cell measurements) were compared.

While fluorescence microscopy was used to measure FRET in EGFR-Grb2 and Epac systems, a first step towards detection in a multi-well reader system was performed with a HeLa cell line expressing a membrane associated green fluorescent protein (HeLa hFR-GPI-GFP, [29].). Part of the cells were incubated with the membrane marker Nile Red (used as an energy acceptor), and the fluorescence lifetime of the donor (GFP) was evaluated as a main FRET parameter for the whole plate.

## 2. Results

### 2.1. Intermolecular FRET between EGFR-CFP and Grb2-YFP

**Figure 2:**
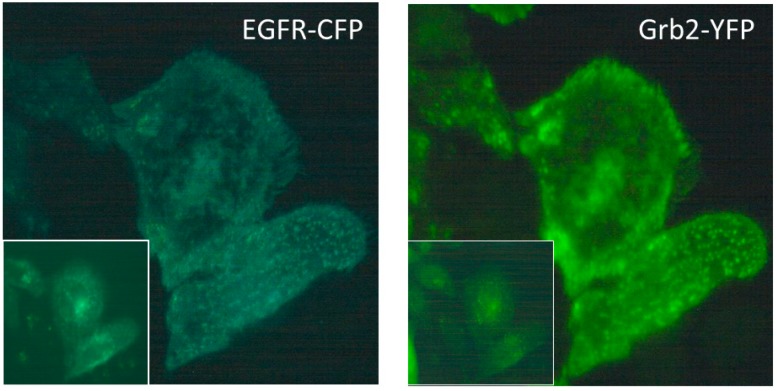
TIR fluorescence images of HeLa cells transfected with EGFR-CFP and Grb2-YFP encoding vectors in the spectral ranges of CFP (450–490 nm) and YFP (λ ≥ 510 nm) emission; excitation wavelength: 420–440 nm; image size: 60 µm × 60 µm. Insets: fluorescence images after epi-illumination of whole cells.

In Figure, two TIR fluorescence images are depicted for HeLa cervix carcinoma cells stably transfected with Grb2-YFP, and transiently transfected with EGFR-CFP without further stimulation. After optical excitation of CFP at 420–440 nm, we recorded blue fluorescence in the spectral range 450–490 nm (Figure 2, left) and green-yellow fluorescence at λ ≥ 510 nm (Figure 2, right). These spectral images result from the bright emission of donor (CFP) and acceptor (YFP) molecules distributed in close vicinity to or within the plasma membrane, respectively. In contrast, fluorescence images in the same spectral ranges after epi-illumination of whole cells (insets in Figure 2) appear blurred and superimposed by a higher background. Therefore, TIR appears to be an appropriate method to selectively measure both EGF-CFP and Grb2-EYFP fluorescence in live cell membranes with significantly reduced cytosolic background, enabling specific and enhanced detection of FRET and FLIM signals.

**Figure 3:**
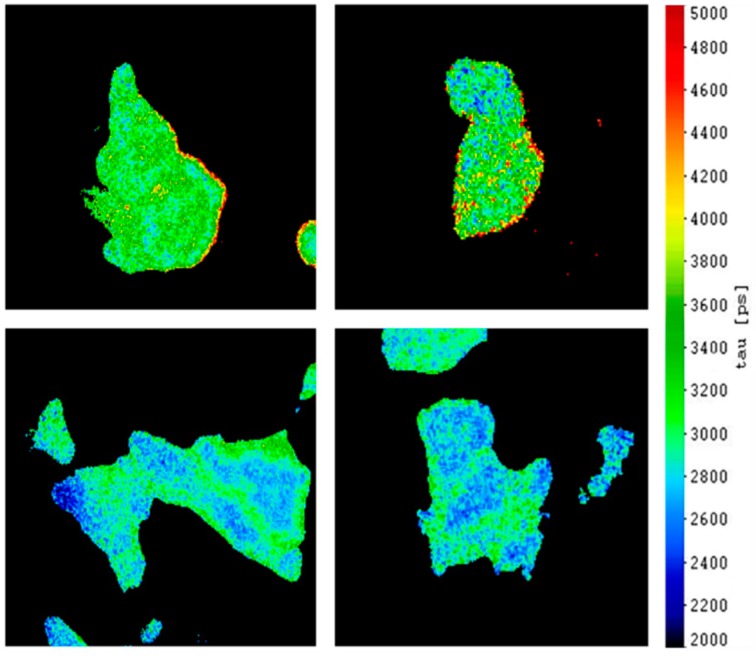
Fluorescence lifetime imaging of EGFR-CFP in HeLa cells upon total internal reflection (TIR) illumination in the absence (top) and in the presence (bottom) of Grb2-YFP including a scale in picoseconds (excitation wavelength: 420–440 nm; detection range: 450–490 nm; image size: 100 µm × 100 µm each).

Figure 3 shows fluorescence lifetime images (FLIM) of the donor (CFP) in the spectral range of 450–490 nm for two object fields of HeLa control cells transiently transfected with EGFR-CFP (top) and two object fields of HeLa cells stably transfected with Grb2-YFP and transiently transfected with EGFR-CFP (bottom). From the attached lifetime scale one can deduce that after co-transfection the fluorescence lifetime is reduced from 3.0 ns–3.4 ns to 2.4 ns–2.8 ns, most probably due to increased deactivation of the excited molecular state of CFP by non-radiative energy transfer to YFP according to Equation (1). A more quantitative evaluation was performed with 13 images of single cells transfected with EGF-CFP in the absence of Grb2-YFP, and 24 images in the presence of Grb2-YFP. The median value (with a median absolute deviation, MAD) was (3.37 ± 0.41) ns in the first and (2.49 ± 0.19) ns in the second case, thus indicating non-radiative energy transfer in the presence of Grb2-YFP, even without stimulation of the cells by EGF. After stimulation (with 50 nM or 100 nM EGF for 15 min) some preliminary measurements showed brightly fluorescent YFP spots at the edges of the cells, possibly due to enhanced FRET in focal adhesions. It remains to be proven whether further changes of the fluorescence lifetime of CFP occur thereby.

### 2.2. Intramolecular FRET within Epac-SH188 Biosensor

**Figure 4:**
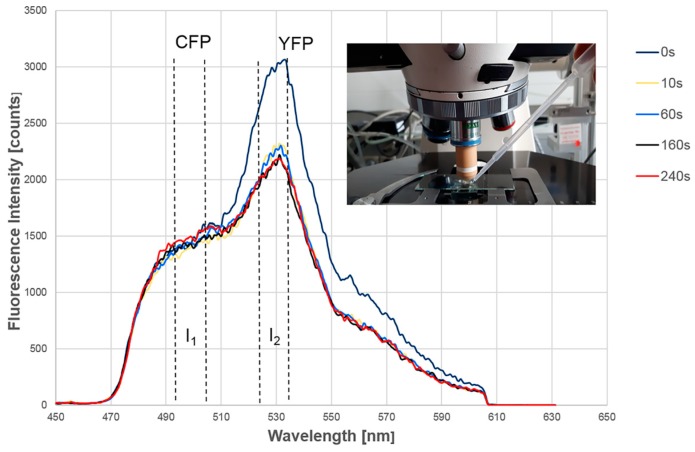
Fluorescence spectra of the Epac-SH188 system expressed in a HEK 293 cell at various times after addition of NECA (1 µM) upon epi-illumination; regions used for evaluation of I_1_ and I_2_ are indicated, and the experimental setup is shown in the inset.

The Epac-SH188 sensor with CFP and YFP as chromophoric groups was expressed in HEK 293 cells as well as in Chinese hamster ovary (CHO-K1) cells with recombinant A2B adenosine receptor. For fluorescence microscopy, cells were kept in an open chamber, and 5′-*N*-ethylcarboxamidoadenosine (NECA; 1 µM) or Forskolin (30 µM) in Tyrode buffer were used for activation and added via a syringe, as depicted in the inset of Figure 4. Hereby, FRET from CFP to YFP was modified.

Figure 4 shows the relevant fluorescence spectra of HEK 293 cells with a YFP band at 530 nm and CFP fluorescence appearing as a shoulder between 480 nm and 510 nm (part of the CFP band at shorter wavelengths was cut off by the long pass filter used for micro-spectral analysis in order to suppress the excitation light efficiently). Following application of NECA (Figure 4) or Forskolin (not shown in this Figure), the YFP band decreased (preferentially within the first seconds) while CFP fluorescence slightly increased during the measuring period of 4 minutes. Then, evaluation of FRET requires determination of the ratio of acceptor (YFP) and donor (CFP) fluorescence. Due to the strong overlap of these spectral bands, we decided to determine fluorescence intensities in a spectral region dominated by YFP fluorescence (525–535 nm; I_2_) and a region with a pronounced contribution of CFP fluorescence (495–505 nm; I_1_). The ratio (I_2_ − I_1_)/(I_2_ + I_1_) was then used as a measure of the efficiency of the energy transfer. This ratio, which is analogue to an algorithm used for polarization spectroscopy, considers the spectral overlap of the two bands as well as background fluorescence and appears, therefore, to be a more robust parameter than the simple intensity ratio I_2_/I_1_. The amount of increase of CFP fluorescence during the experiment varied between individual cells, but it was generally less pronounced than the decrease of YFP fluorescence. This may be related to the low fluorescence quantum yield of CFP in comparison to YFP (although some efforts to increase this quantum yield have been reported in the literature [30]).

**Figure 5:**
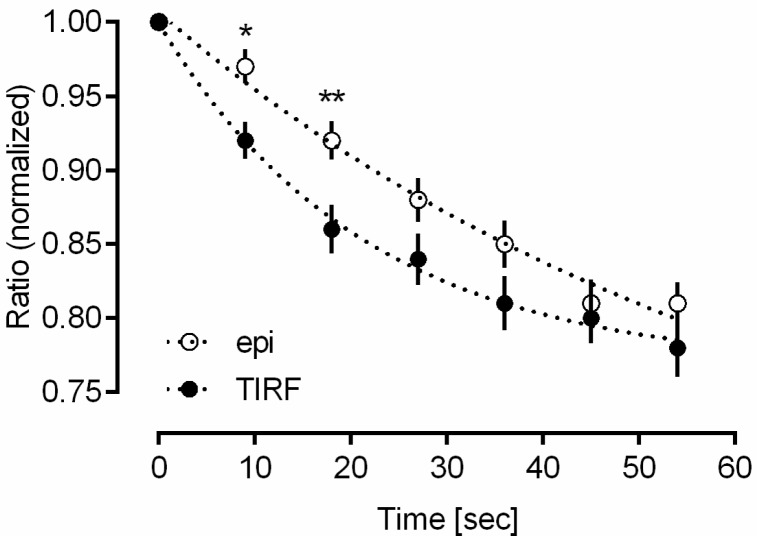
Ratio (I_2_ − I_1_)/(I_2_ + I_1_) in HEK 293 cells as a measure of energy transfer. Means ± SEM (*n* = 10) upon epi-illumination and TIR illumination over a period of one minute in intervals of 9 s after application of NECA (levels of significance: * *p* < 0.05 and ** *p* < 0.01 for comparison of epi- and TIR-illumination). All starting values were normalized to 1. Data points were fitted using a single-exponential decay function (Graphpad Prism).

The ratio (I_2_ − I_1_)/(I_2_ + I_1_) was evaluated for 10 cells in each case as mean ± standard error of the mean (SEM) upon illumination of whole cells (epi-illumination) or selective illumination of the plasma membrane and adjacent cellular sites (TIR illumination) over a period of one minute in intervals of 9 s after application of NECA. All starting values were normalized to 1. Figure 5 shows a decrease in the ratio over the whole period, but this decrease occurred more rapidly in TIR than in the epi-configuration with significant differences after 9 s and 18 s (the strong initial decrease in acceptor fluorescence within 10 s upon epi-illumination, as depicted in Figure 4, was a rather unique result). This proves a more rapid reaction towards NECA in TIR experiments in close proximity to the plasma membrane in comparison with whole cells.

**Figure 6:**
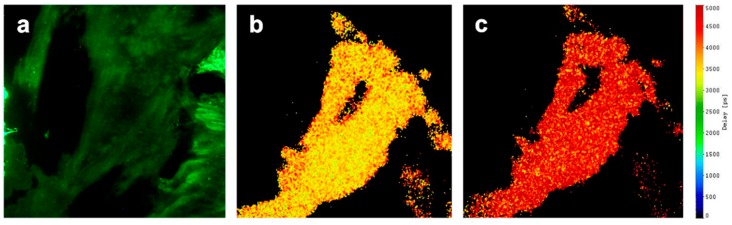
(**a**) TIR fluorescence intensity of the Epac sensor in CHO-K1 cells in the spectral range λ ≥ 470 nm, and (**b**,**c**) fluorescence lifetime of the donor CFP measured upon TIR excitation in the spectral range of 450–490 nm at 0 s (**b**) and 10 s (**c**) subsequent to addition of Forskolin (image size; 100 µm × 100 µm); scale in picoseconds.

In contrast to the HEK 293 cells, which only showed a few focal contacts with the glass substrate, CHO-K1 cells showed broader contact areas, so that this cell line appeared to be more appropriate for TIR imaging, including FLIM. This is documented in Figure 6a, showing the TIR fluorescence intensity of CHO-K1 cells expressing the Epac-SH188 sensor in the spectral range λ ≥ 470 nm (including both CFP and YFP fluorescence). The fluorescence lifetime of the donor CFP measured upon TIR excitation in the spectral range of 450–490 nm is depicted in Figure 6 for a cluster of three cells at 0 s (b) and 10 s (c) subsequent to addition of Forskolin. This lifetime was prolonged from about 3.00 ns to 4.00 ns and indicates a rapid decrease in FRET efficiency in close proximity to the plasma membrane corresponding to Equation (1).

### 2.3. Intermolecular FRET in a HeLa hFR-GPI-GFP Test System Using Nile Red as an Energy Acceptor

**Figure 7:**
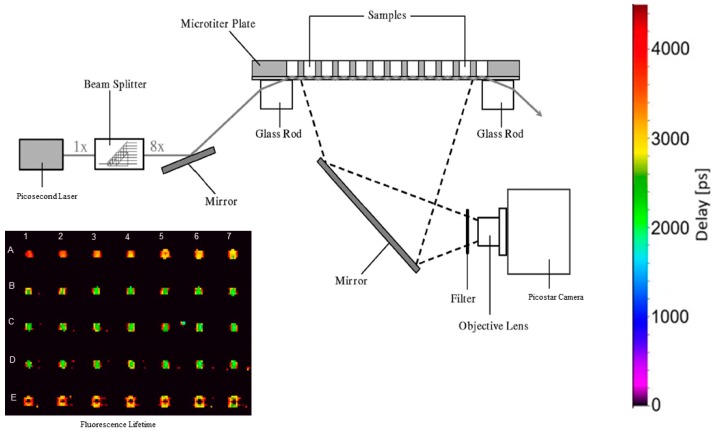
TIR fluorescence lifetime reader for a 96-well microtiter plate including scale. Inset: Fluorescence lifetimes of HeLa hFR-GPI-GFP cells prior to (arrays A,E) and subsequent to (arrays B,C,D) incubation with 30 µM Nile Red for 10 min.

In the TIR microscope emission maxima of the membrane associated fluorophores GFP and Nile Red were registered around 510 nm and 630 nm, respectively, as further documented in [31]. In addition, a reduction in the fluorescence lifetime of the donor (GFP) from 2.2 ± 0.25 ns to about 1 ns was detected after incubation with the energy acceptor Nile Red. We then tested FRET imaging in a multi-well fluorescence reader upon simultaneous TIR excitation of up to 96 individual wells. As depicted in Figure 7 and further reported in Section 4.3, a picosecond laser beam was split into eight beams, each of which was totally reflected on 12 individual wells. The inset of Figure 7 documents the different fluorescence lifetimes of GFP in 35 wells of the microtiter plate prior to (arrays A,E) and subsequent to incubation with Nile Red (arrays B,C,D). In the latter case the fluorescence lifetime decreased from about 3.00 ns to 2.20 ns due to non-radiative energy transfer (FRET).

## 3. Discussion

This manuscript reports on intramolecular and intermolecular FRET as well as its possible changes upon addition of pharmaceutical agents. Of particular interest are measurements with TIR illumination, since plasma membrane associated molecules, e.g., EGFR-CFP, Grb2-EYFP and their interactions, are recorded selectively. However, TIR experiments also appear to be applicable to molecules inside the cells, if their expression level is high enough, and if part of them are located close to the plasma membrane, since not only fluorescence of these molecules, but also the background is reduced in comparison with measurements of whole cells. This is described in the present manuscript for the Epac-SH188 sensor of cyclic AMP. In case of a homogeneous distribution of fluorophores, a rough rule is that upon TIR illumination the fluorescence ratio and whole cell illumination corresponds to the ratio of the penetration depth of the evanescent wave (typically 70–200 nm) and the thickness of a cell monolayer (typically 5–10 µm), which amounts to a factor of 1/30–1/100. Therefore, cameras of high sensitivity, e.g., sCMOS or electron multiplying CCD cameras, are advantageous for high quality TIR imaging. FRET can be assessed by spectroscopic measurements (applied to the Epac system. for example) or by fluorescence lifetime detection. Spectroscopic measurements are based on detection of fluorescence intensity and affected by various factors, e.g., inhomogeneous illumination, spectral overlap or degradation of fluorophores upon addition of chemical agents, whereas fluorescence lifetime measurements may depend on molecular conformation (without FRET [32,33]), but otherwise are directly related to the energy transfer rate according to Equation (1). In addition, fluorescence lifetime imaging (FLIM) does not need any correction for different fluorescence quantum yields of donor or acceptor molecules. Therefore, in all parts of this project (EGFR-Grb2 interaction, Epac-SH188 sensor, interaction of GFP with Nile Red) shortening of the fluorescence lifetime of donor molecules are regarded as evidence for FRET.

TIR measurements also permit the evaluation of differences between membrane associated and intracellular reactions. In case of the Epac-SH188 sensor, a decrease in FRET efficiency upon addition of NECA or Forskolin seems to occur more rapidly within, or in close proximity to the plasma membrane than in the inner parts of the cell. This is well documented in Figure 5, however, up to now it is not clear whether the reaction is favored by any membrane specific properties, or whether NECA and Forskolin just reach membrane proximal sites more rapidly. Also, an intracellular translocation of the Epac sensor upon application of NECA or Forskolin should be considered. This effect would be independent of FRET and might explain the different behaviors close to the plasma membrane and in the inner parts of the cell. However, in this case the fluorescence intensities of CFP and YFP should change in the same way, i.e., increase or decrease simultaneously. In contrast, a slight increase of CFP fluorescence was always concomitant with a decrease of YFP fluorescence, which is typical for a decrease in FRET, and which is also described for the Epac sensor in the literature [27]. Here, the ratio of YFP and CFP fluorescence depended on the kind of stimulation, the concentration of the agents and the time of interaction, but the ratio always decreased due to the reduction in FRET efficiency. Finally, we should emphasize that we always worked with low light exposure (doses below 5 J/cm^2^), where photobleaching or phototoxic cell damage was negligible [34].

For drug screening experiments, FRET imaging in a multi-well fluorescence reader (e.g., a 96-well microtiter plate) appears to be of high relevance. Fluorescence lifetime [35,36] as well as TIR [37] readers have been reported previously. However, in a TIR-based FRET reader system only individual wells of a microtiter plate have been studied so far [38]. Therefore, simultaneous TIR-FRET imaging of multiple wells, as reported above and depicted in Figure 7, remains a challenge for further experiments. The inset of Figure 7 documents the different fluorescence lifetimes of a membrane associated GFP in individual wells of the microtiter plate prior to (arrays A, E), and subsequent to incubation with Nile Red (arrays B, C, D). However, application of TIR-FRET imaging needs sufficiently high transfection rates of membrane associated proteins as well as highly sensitive time resolving cameras (e.g., Picostar HR12 image intensifier coupled to a cooled ICCD camera, LaVision, Göttingen, Germany) to detect comparably low fluorescence signals, and a detection system that uses a moderate aperture (to avoid optical distortion). A problem of time-resolved FRET experiments may arise from the fact that the excitation light reaches individual wells of the microtiter plate with time delays of up to one nanosecond between one another. This problem can only be overcome if FLIM is evaluated separately for individual parts of the microtiter plate, or if the number of evaluated wells remains limited, e.g., 7 × 5, as depicted in the inset of Figure 7.

After successful application of our TIR fluorescence reader for energy transfer measurements between GFP and Nile Red, it is now our goal to use this reader for FRET measurements between EGFR-CFP and Grb2-YFP. First test experiments after stimulation by EGF have been performed, resulting in brightly fluorescent YFP spots at the edges of the cells, possibly due to enhanced FRET in focal adhesions. Further applications with inhibitors of FRET (e.g., EGFR phosphorylation inhibitors such as AG1478) [26,39,40] are scheduled with a view to designing a test system for pharmacological agents. This, however, requires monolayers of cells with sufficiently high transfection rates (≥50%) for reliable evaluation by the TIR-FRET reader (without visual control).

## 4. Materials and Methods

### 4.1. Recombinant Cell Lines and Cell Culture Conditions

HeLa Grb2-YFP cervix carcinoma cells were cultivated at 37 °C, 5% CO_2_, in RPMI 1640 medium supplemented with 10% FBS, 1% penicillin/streptomycin, 1% G418 and stable glutamine. Non-transfected (control) HeLa cells were cultured under the same conditions without G418. For TIR, fluorescence spectroscopy and fluorescence lifetime recordings, 500 cells/mm^2^ (Hela control and Hela Grb2-YFP) were seeded on glass object slides. Twenty-four hours after seeding, cells were transiently transfected with plasmid DNA (EGFR-CFP, 1 μg/object slide). Object slides were rinsed 48 h after transfection with Earl’s balanced salt solution (Sigma-Aldrich, Germany), and cells were imaged within the microscope.

HEK 293 cells and CHO-K1 cells transfected with Epac-SH188 + A2B adenosine receptor were cultivated at 37 °C, 5% CO_2_, in DMEM high glucose medium supplemented with 10% FBS, 1% non-essential amino acids, 1% Na-Pyruvat and 2% Hepes. For TIR, fluorescence spectroscopy and fluorescence lifetime imaging, 400 cells/mm^2^ were seeded on glass object slides with three removable chambers (Ibidi, Martinsried, Germany). Cells were washed with Tyrode-Buffer and imaged on the microscope. Incubation with NECA (1 µM) and Forskolin (30 µM) occurred directly on the object slide after measuring the reference spectra. Spectra were recorded in intervals of 9 s, and FRET efficiency was evaluated in each case.

HeLa hFR-GPI-GFP cells were cultivated under the same conditions as HeLa Grb2-YFP cells. Prior to experiments, Nile Red was added for 10 min. at a concentration of 30 µM in phosphate buffered saline (PBS) after 100-fold dilution of a 3 mM acetone stock solution. After incubation with Nile Red, cells were rinsed with PBS.

### 4.2. Fluorescence Microscopy

Microscopy experiments were performed with a super-continuum fiber laser (SuperK, NKT Photonics, Birkeröd, Denmark) emitting a series of pulses of 5 ps duration with a repetition rate of 78 MHz. Emitted laser light was selected in the spectral range of 420–440 nm (corresponding to the absorption band of CFP) by a SuperK VARIA optical tuning filter (NKT Photonics, Birkeröd, Denmark). The laser was adapted to a fluorescence microscope (Axioplan 1, Carl Zeiss Jena, Jena, Germany) with either a multimode fiber and a dichroic mirror (reflecting light at wavelengths λ ≤ 460 nm) for epi-illumination or with a single mode fiber (NKT Photonics, Denmark) for illumination by an evanescent electromagnetic field (penetration depth around 130 nm) with a specific device for total internal reflection (TIR) fluorescence microscopy [8]. In the first case, whole cells were illuminated whereas in the second case, selective illumination of the plasma membranes and adjacent cellular sites occurred. Measurements were performed in an open chamber with a 63×/0.90 water immersion objective lens. For fluorescence spectroscopy, a long pass filter transmitting light above 470 nm was used. Fluorescence spectra of single cells were recorded with a polychromator and an image intensifying detection unit (IMD 4562, Hamamatsu Photonics, Ichino-Cho, Japan) fixed on top of the microscope. For fluorescence lifetime imaging (FLIM), an interference filter for 450–490 nm and a time-gated image intensifying CCD camera system (Picostar HR 12; LaVision, Göttingen, Germany) with a time resolution of 200 ps were used, as described in detail elsewhere [41]. Fluorescence lifetime images of individual cells were calculated from time-gated images recorded within 200 ps when the time gate was shifted over an axis of 8 ns. Conventional color images were recorded with a commercially available Canon reflex camera.

### 4.3. TIR Fluorescence Multi-Well Reader

A fluorescence multi-well reader for simultaneous TIR illumination of up to 96 samples in a microtiter plate with a custom-made glass bottom (Optiwhite) has been described previously [37]. Compared to [37], the argon ion laser was replaced by the super-continuum laser with picosecond pulses and an excitation band of 430–450 nm (see Figure 7). A long pass filter for λ ≥ 495 nm and an interference filter for (497 ± 27) nm were used to select donor fluorescence for FRET experiments with the image intensifying camera system reported above. In contrast to fluorescence microscopy a camera objective lens of *f’* = 25 mm was used, and a time gate of 700 ps was shifted over an axis of 8 ns. Broadening of the time gate compared to fluorescence microscopy (200 ps) was necessary to compensate for some time shift between optical excitation of the individual wells.

### 4.4. Statistics

Significance testing was performed using GraphPad Prism 6.0 for Windows. Differences were considered significant with *p* < 0.05 for multiple t-test followed by the Holm-Sidak method for multiple comparison tests. All values are given as the means ± standard error of the mean (SEM) if not otherwise stated.

## 5. Conclusions

We conclude that measurements of FRET between fluorescent proteins under TIR illumination are appropriate to detect molecular interactions or changes of molecular conformation due to pharmaceutical agents in close proximity to the plasma membrane. Fluorescence from inside the cells as well as background signals arising from the supernatant are efficiently suppressed, which makes this method very specific and sensitive. This was proven for three applications using a fluorescence microscope or a multi-well TIR reader system applied for simultaneous measurement of a larger number of samples, e.g., cells expressing EGFR-CFP and Grb2-YFP. FRET efficiency was assessed by detection of fluorescence intensity of donor and acceptor molecules or by nanosecond fluorescence lifetime measurement of the donor. Evaluation appears to be easier and more reliable in the latter case, however, in a multi-well fluorescence reader a time shift between excitation of individual samples should be taken into account.
